# Liver X receptors and estrogen receptor β, two players in a rare subtype of NSCLC

**DOI:** 10.7150/ijbs.85164

**Published:** 2023-05-29

**Authors:** Wanfu Wu, Mozhgan Sarhadi, Xiaoyu Song, Jingling Xue, Yubing Dai, Jan-Ake Gustafsson

**Affiliations:** 1 Center for Nuclear Receptors and Cell Signaling, Department of Biology and Biochemistry, University of Houston, Houston, TX 77204, USA.; 2 Department of Medical Microbiology, School of Basic Wuhan University, Wuhan, Hubei 430071, China.

**Keywords:** nuclear receptor, lung cancer, macrophages, T cell, cigarette smoke

## Abstract

Liver X receptors (LXRαβ) play essential roles in the maintenance of the normal functions of macrophages, in modulation of immune system responses and cholesterol homeostasis. We have reported that LXRαβ^-/-^ mice develop squamous cell lung cancer. We now report that those LXRαβ^-/-^ mice, which live to 18-months of age, spontaneously develop a second type of lung cancer resembling a rare subtype of NSCLC (TTF-1 and P63-positive). The lesions are characterized as follows: a high proliferation rate; a marked accumulation of abnormal macrophages; an increase in the number of regulatory T cells; a remarkably low level of CD8^+^ cytotoxic T lymphocytes; enhanced TGFβ signaling; an increased expression of matrix metalloproteinases accompanied by degradation of lung collagen; and a loss of estrogen receptor β (ERβ).

Because NSCLC is associated with cigarette smoking, we investigated the possible links between loss of LXRαβ and CS. A Kaplan-Meier Plotter database revealed reduced expression of LXRαβ and ERβ was correlated with low overall survival (OS). Thus, reduction of LXRαβ expression by cigarette smoking may be one mechanism through which CS causes lung cancer. The possibility that maintenance of LXRαβ and ERβ signaling could be used in the treatment of NSCLC needs further investigation.

## Introduction

Cigarette smoke (CS) is a well-known risk factor for lung disease. Numerous compounds from CS can adversely affect the lungs 1. CS induces tissue damage around the airways and alveoli and has widespread effects in the lung which contribute to lung cancer development and progression. These include: alteration of gene expression with induction of oncogene expression and blocking the expression of tumor-suppressor genes 2; induction of xenobiotic and redox-regulating genes 2; alteration in intracellular signaling pathways particularly those involved in nicotine signaling through adrenergic receptors; compromised immune responses 3-6; increase in oxidation of lipids and proteins; induction of endoplasmic reticulum (ER) stress; and deregulation of the normal ceramide metabolism 7, 8.

Innate immunity, surfactant/lipid processing and iron homeostasis within the respiratory tract 9 are all regulated by lung macrophages. Macrophage functions are markedly influenced by CS. CS alters macrophage polarization 10, 11, increases recruitment 12, changes phagocytosis and bacterial killing 13, promotes formation of reactive oxygen species (ROS), increases proteinase/anti-proteinase release 14, and disrupts iron and lipid homeostasis 15, 16. CS-induced macrophage dysfunction leads to inflammation and resultant destruction/remodeling which increases susceptibility to development of CS-induced lung diseases 17. CS contributes to the development of lung cancer with both pro-inflammatory and immunosuppressive effects 18, 19. CS was reported to induce ADAM12^+^ CTLA4^+^ Tregs (a unique subset of tumor-specific activated Tregs) which interact with exhausted T cells in the tumor immune environment of lung cancer 20.

Liver X receptors (LXRαβ), LXRα (NR1H3) and LXRβ (NR1H2) are important regulators of cholesterol, fatty acid, and glucose homeostasis. LXRαβ are highly expressed in macrophages where they regulate expression of genes related to of cholesterol metabolism and immunity 23. LXRαβ in macrophages are activated when cholesterol is high and this results in suppression of inflammatory genes 24, 25, promotion of cellular efflux of cholesterol by inducing expression of the ATP-binding cassette transporters ABCA1 and ABCG1 and facilitation cholesterol transport by inducing Apo E (apolipoprotein E) 26. LXRαβ promote expression of the Trem2 gene which suppresses inflammation in foam cells in atherosclerotic plaques 27 while deficiency of LXRαβ in myeloid cells caused an increase in atherosclerosis with increased monocyte infiltration, foam cell formation, and plaque inflammation.

Our previous studies have demonstrated that in mice lacking LXRβ, macrophages and microglia are overactive with resultant damage to the cochlear and dopaminergic neurons 28, 29. LXRαβ are expressed in the lung epithelium both mice and humans 30, 31 and in LXRαβ knock out mice there was accumulation of macrophages (foam cells) in the lung, spleen and arterial wall 32-34 and spontaneous development of peripheral squamous cell lung cancer 34.

CS has been shown to affect the expression of LXRαβ in the lung but whether or not this effects is beneficial or adverse is still not clear: CS has been shown to down-regulate LXRα, ABCA1, and ABCG1 expression in both lungs and cultured alveolar macrophages from rats while activating LXR pathways by Allyl isothiocyanate (AITC) improved chronic obstructive pulmonary disease (COPD) rat model by reducing inflammatory response and promoting reverse cholesterol transport 35. On the other hand, upon exposure to cigarette smoke, LXR activation caused oxidation of surfactant, an adverse effect 36.

In the present study we investigated the lungs of 18-month-old LXRαβ^-/-^ mice and their WT littermates. We found spontaneous development of lesions resembling TTF-1/P63-positive non-small-cell lung cancer (NSCLC), a rare subtype of lung cancer. Our results support a protective role for LXR against the development of lung cancer.

## Materials and Methods

### Animals and tissue preparation

Five 4-mo-old, 12-mo-old, and 18-mo-old male C57BL/6 LXRαβ^-/-^ mice and five WT male littermates were used for this study. The generation of LXRαβ^-/-^ mice has been reported previously 37. The mouse studies were approved by the local Animal Experimentation Ethics Committee for animal experimentation (University of Houston animal protocol 09-036). All experimental protocols to the National Institutes of Health Guidelines for the Care and Use of Laboratory Animals were adhered. Mice were fed with standard rodent chow and housed in a room of standard temperature (22 ± 1°C) with a regular 12-h light, 12-h dark cycle and given free access to water. All mice were terminally anesthetized by CO2 and transcardially perfused with PBS followed by 4% paraformaldehyde (in 0.1 M PBS, pH7.4). The lungs were dissected and postfixed in 4% paraformaldehyde overnight at 4ºC. After fixation, one lung was processed for paraffin sections (5 μm) for hematoxylin and eosin staining, immunohistochemistry, Masson's trichrome stain, and immunofluorescence staining. The other lung was processed for frozen sections.

### Immunohistochemistry (IHC)

Paraffin-embedded slides were dewaxed in xylene, rehydrated in different concentration of ethanol, and then processed for antigen retrieval with 10 mM citrate buffer (pH 6.0) in a Lab Vision PT module (Thermo Scientific) at 97°C for 12 min. The cooled slides were then immersed in 0.5% Triton for 10 min and washed with 1X PBS for 3 times. To quench endogenous peroxidase, the slides were treated in a buffer composed of 50% (vol/vol) methanol and 0.3% (vol/vol) H_2_O_2_ for 30 min and then unspecific binding was blocked by incubating the slides with 3% (vol/vol) BSA in PBS for 30 min. Sections were then incubated with anti-Ki67 (1:1000; Abcam, ab15580), anti-PCNA(1:10000; Abcam, ab29), anti-P40 (1:200; Abcam ab203826), anti-P63 (1:1000; Abcam, 124762), anti-TTF-1[SP141] (1:200; Abcam ab227652), anti-TTF-1[EPR8190-6] (1:1000; Abcam, ab133638), anti-CD68 (1:500; Abcam, ab125212), anti-pSMAD2 (1:100; Chemicon, ab3849), anti-SMAD4 (1:100; Abcam, ab40759), anti-PTEN (1:100; Abcam, ab170941), anti-pAKT (1:100; Abcam, ab40759), anti-Wnt3a (1:100; Abcam, ab198220), anti-β Catenin (1:100; Abcam, ab32572), anti-pEGFR (1:500; Abcam, ab40815), anti-E-cadherin (1:1000; Abcam, ab231303), anti-vimentin (1:1000; Abcam, ab92547), anti-ERβ (1:100; in house ERβ antibody mapping the C-terminus part), anti-MMP12 (1:100; Novus Bio, NBP2-67344), anti-MMP8 (1:100; Abcam, ab81286), anti-MMP14 (1:100; Abcam, ab73877), anti-FOXP3 (1:100; Abcam, ab54501), anti-CD3 (1:500; Santz Cruz, sc-1127), anti-CD8 (1:500; Abcam, ab237723) at 4°C after blocking nonspecific binding in 3% BSA. BSA replaced primary antibodies in negative controls. The images for negative control were presented in supplementary materials (Fig. S1). After washing, sections were incubated with biotin-labeled secondary antibody for 1h at room temperature. After 3 washes, sections were incubated with ABC (Vector lab, PK-4000) for 1h at room temperature. Sections were then developed by 3, 3-diaminobenzidine tetrahydrochloride as the chromogen. We stained every fifth slide from 25 consecutive slices i.e., five slices from each mouse. The Keyence microscope (BZ-X810) was used to take the images and count the cell numbers.

### Immunofluorescence double staining

For immunofluorescence double staining, quenching of endogenous peroxidase and blocking of endogenous biotin were omitted. Sections were incubated with anti-TTF1 SP141 (1:200; Abcam ab227652) and anti-P40(1:1000; a gift from Dr. Philip J Coates, Masaryk Memorial Cancer Institute, Czech Republic); or with anti-MMP12 (1:100; Novus Bio, NBP2-67344) and anti-F4/80 (1:100; Bio-Rad, MCA497GA); or with anti-IL-1β (1:100; abcam, ab283818) and anti-F4/80( 1:100; Bio-Rad, MCA497GA); or with anti-Arg1(1:100; Santa Cruz, sc-18351) and anti-F4/80 (1:1000; Bio-Rad, MCA497GA); or with anti-IL-1β (1:100; abcam, ab283818) and anti-Arg1(1:100; Santa Cruz, sc-18351). Primary antibodies were detected with donkey anti-rabbit FITC (1:400; Invitrogen, A21206) and donkey anti-mouse 594 (1:400; Invitrogen, A21203) or donkey anti rabbit FITC (1:400; Invitrogen, A21206) and donkey anti-rat 594 (1:400; Invitrogen, A21209); donkey anti rabbit FITC (1:400; Invitrogen, A21206) and donkey anti-goat Cy3 (1:400; Jackson ImmunoResearch, 05-165-147). Samples were covered with medium containing with 4', 6'-diamidino-2-phenylindole (DAPI) (Vector) for cell nuclei visualization. The Keyence microscope (BZ-X810) was used to take the images.

### Masson's Trichrome Staining

The Masson's Trichrome Staining has been previous described 38. Briefly sections were re-fixed in Bouin's solution for 1 hour at 56 C after dewaxed and rehydrated. Then sections were stained in Weigert's iron hematoxylin working solution for 10 minutes followed by stained in Biebrich scarlet-acid fuchsin solution for 15 minutes then differentiated in phosphomolybdic-phosphotungstic acid solution for 15 minutes. Sections were transferred to aniline blue solution and stained for 10 minutes. After differentiation in 1% acetic acid solution for 5 minutes, sections were dehydrated and mounted. The Keyence microscope (BZ-X810) was used to take the images.

### Oil Red O staining

According to the manufacturer's instruction frozen sections were stained with Oil Red O stain Kit (American MasterTech., KTORO). Slides were mounted with glycerine jelly mounting medium. The Keyence microscope (BZ-X810) was used to take the images.

### RNA Sequencing

The inferior lobe of right lungs of three 12-month-old WT mice and three 12-month-old LXRαβ^-/-^ mice were used. Detailed information can be found from our published paper 34.

### Kaplan-Meier survival analysis

Kaplan Meier plotter database was used (https://kmplot.com/analysis/) for the analysis. Briefly, for LXRα (NR1H3), LXRβ(NR1H2) and ERβ(ESR2), first gene name was input, then squamous cell carcinoma was included in Histology, at last exclude those who never smoked was selected in Smoking history.

### Data analysis

Data are expressed as mean ± SD; statistical comparisons were made by using a one-way ANOVA followed by Newman-Keuls post-hoc test. Student *t* test was used for two group comparison. P<0.05 was considered to indicate statistical significance.

## Results

### The spontaneous development of atypical peripheral SCC-like lesions in 18-month-old LXRαβ^-/-^ mouse lungs

**Figure 1:**
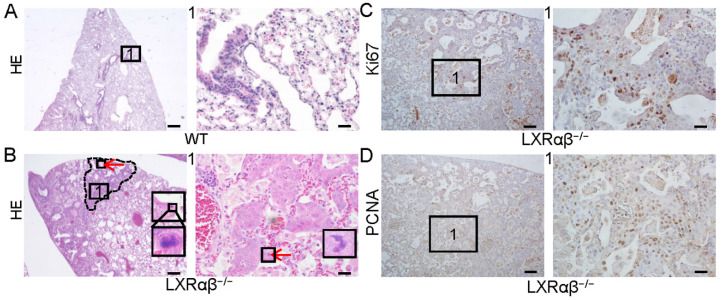
** Non-small-cell lung cancer-like lesion in LXRαβ^-/-^ mouse lung.** HE staining showed normal structure of 18-month-old WT mouse lung (**A**). There were NSCLC-like lesions (**B**) in 18-month-old LXRαβ^-/-^ mouse lung. Inserted high magnification pictures in **B** showed abnormal mitosis. No obvious intercellular bridges were found by HE staining. The characteristics of the HE staining preferred the diagnosis as atypical LUSC. Positive immunoreactivity for Ki67 (**C**) and PCNA (**D**) indicated these cells were highly proliferating. Red arrow indicated abnormal mitosis. PCNA: proliferating cell nuclear antigen. (Scale bars in A and B, 500 μm and 50 μm; Scale bars in C-D, 200μm and 50 μm)

In a previous study we reported spontaneous peripheral squamous cell lung cancer-like lesion in 14-month-old LXRαβ^-/-^ mouse lungs 34. Although these mice had to be euthanized at 14 months of age because of respiratory distress, some mice survived until they were 18 months old. The lungs of these mice (n=5) were compared with those of 18-month-old WT littermates (Fig. 1 A, B). Atypical peripheral SCC-like lesions were identified by H&E staining (Fig. 1 B) with atypical mitosis, very mild keratinization and absence of intercellular bridges in 3 mice. The cells in the lesion had a high proliferation rate as measured by positive staining for Ki67 (Fig. 1 C) and proliferating cell nuclear antigen (PCNA) (Fig. 1 D).

### Co-expression of TTF-1 and P63 in the cells resembling TTF-1/P63-positive NSCLC

**Figure 2:**
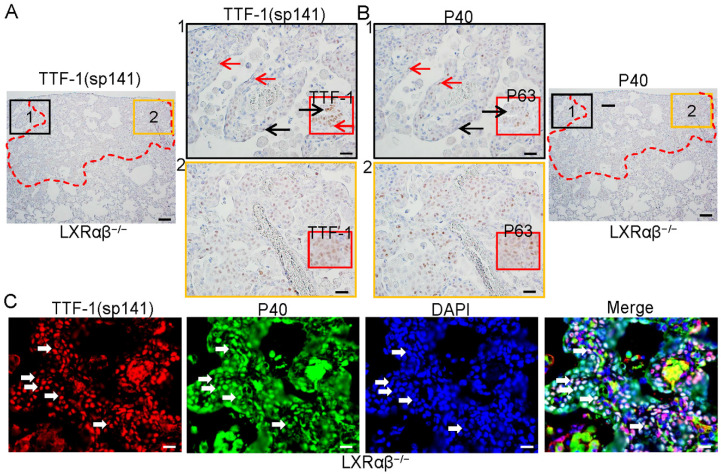
** The co-expression of TTF-1 and P40 in the cells of the cancer-like lesion in 18-month-old LXRαβ^-/-^ mouse lung.** TTF-1[SP141] and P40 staining on serial sections showed co-expression of TTF-1 and P40 in the cells of peripheral bronchial basal layer adjacent to the lesion (red arrows), TTF-1 positive and P40 negative in the cells of luminal layer (black arrows) (**A1**, **B1**). Insert pictures in A1 and B1 (TTF-1[EPR8190-6] and P63 staining respectively) showed basal cells are both TTF-1 positive and P63 positive (red arrow), luminal cells were TTF-1 positive and P63 negative (black arrow). TTF-1[SP141] and P40 were co-expressed in the cells of the lesion (**A2**, **B2**). Insert picture in A2 was TTF-1[EPR8190-6] staining and insert picture in B2 was P63 staining. Double immunofluorescence staining for TTF-1 (red), P40 (green) and DAPI (blue) confirmed the co-expression of TTF-1 and P40 in the cells (white arrows) (**C**). (Scale bars in A and B, 200 μm and 50 μm. Scale bars in C, 30 μm.)

To characterize the cells in these lesions, immunochemistry (IHC) with antibodies against TTF-1(sp141), TTF-1(EPR8190-6), P40 (delaNp63) and P63 were used. Stainings were done on serial sections. The cells in the basal layer of the terminal bronchial adjacent to the lesion were both TTF-1 and P40-positive as were the cells in the lesion (red arrows in Fig. 2 A1, B1). Cells in luminal layer were TTF-1-positive, but P40-negative (black arrows in Fig. 2 A1, B1). The cells in the lesion were both TTF-1(sp141)-positive and P40-positive (Fig. 2 A2, B2). Insert pictures in A1 and A2 are TTF-1 (EPR8190-6) staining, in B1 and B2 were P63 staining. The co-expression of TTF-1 and P40 was further confirmed by immunofluorescence double staining (Fig. 2 C). Dysplastic lesions with TTF-1/P40-positive cells with obvious lower proliferative rate than cancer-like lesions were also found in five LXRαβ^-/-^ mouse lungs (Fig. S2). In one LXRαβ^-/-^ mouse, both a typical peripheral SCC-like lesion and a TTF-1/p63-positive lesion were found in the same lung. The typical peripheral SCC-like lesion was identified by keratinization, intercellular bridges and atypical mitosis (Fig. S3 Ai). The cells were TTF-1-negative or very weakly positive (Fig. S3 Bi), P40-positive (Fig. S3 Ci), and highly proliferating (Fig. S3 Di). However, cells in the TTF-1/P40-positive lesions showed atypical mitosis without keratinization or intercellular bridges (Fig. S3 Aii), TTF-1-positive (Fig. S3 Bii), P40-positive (Fig. S3 Cii), and high expression of proliferation markers (Fig. S3 Dii).

### Accumulation of activated macrophages with lipid droplets in LXRαβ^-/-^ mouse lung

**Figure 3:**
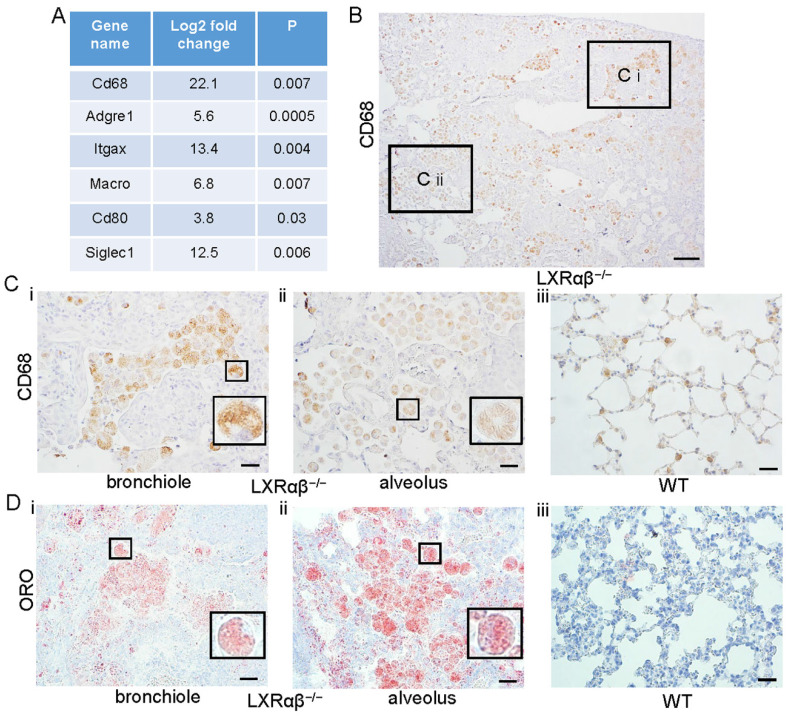
**Increased activated macrophages with neutral lipids and lipid droplets accumulation in LXRαβ^-/-^ mouse lung.** Some genes for macrophage were upregulated from 4-fold to 22-fold by RNA-seq (**A**). CD68 staining showed enormous macrophages in LXRαβ^-/-^ mouse lung (**B**) including in bronchioles (**Ci**) and alveoli (**Cii**). CD68 staining showed normal macrophages in WT mouse lung (**Ciii**). Insert pictures in Ci and Cii were higher magnification for two macrophages containing cholesterol clefts in cytoplasm. Oil-Red-O staining demonstrated neutral lipids and lipid droplet accumulation in macrophage in bronchioles (**Di**) and alveoli (**Dii**) in LXRαβ^-/-^ mouse lung. Macrophages in WT mouse lung were Oil-Red-O negative (**Diii**). Insert pictures in Di and Dii were higher magnification for two macrophages. (Scale bars in B, 200 μm; Scale bars in C and D, 50 μm.)

Comparison of gene transcripts from lungs of 12-mo-old LXRαβ^-/-^ mice with 12-mo-old WT mice revealed that macrophage genes were significantly upregulated (Fig. 3 A). There was a marked increased accumulation of CD68-positive macrophages, including in bronchioles and alveoli (Fig. 3 B). Compared with macrophages in the lung of WT mice (Fig. 3 Ciii), macrophages from LXRαβ^-/-^ mouse had had larger cell bodies and there were cholesterol clefts in cytoplasm (Fig. 3 Ci, Cii). Neutral lipids and lipid droplet accumulation in macrophages in the lung of LXRαβ^-/-^ mice was further confirmed by positive staining for Oil-Red-O (Fig. 3 Di, Dii). Macrophages in WT mouse lung had no detectable lipid accumulation (Fig. 3 Diii).

### The enhanced TGFβ signaling in the lesions

**Figure 4:**
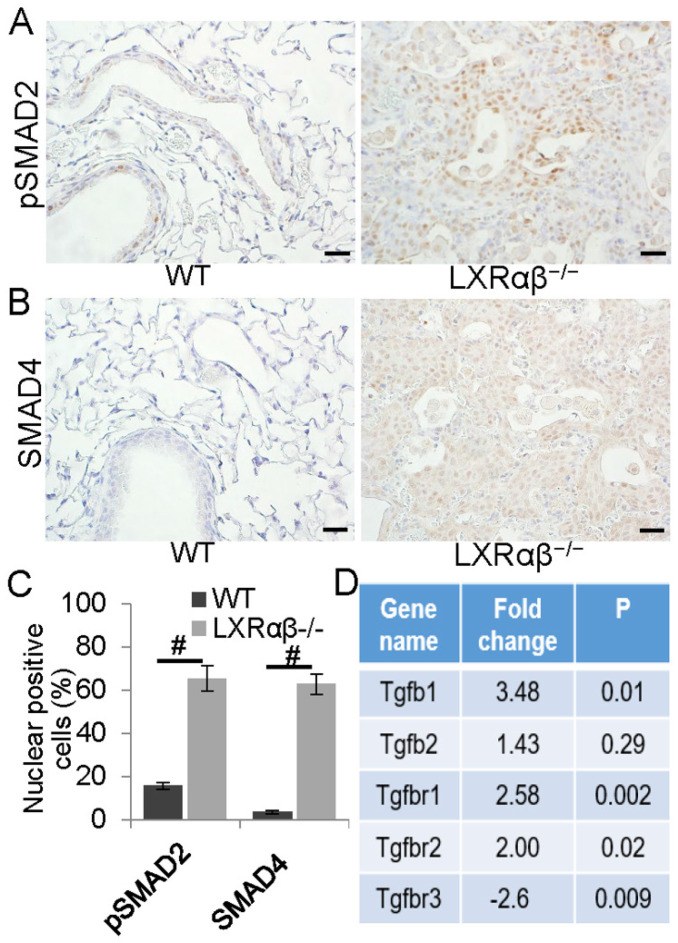
** Enhanced TGFβ signaling pathway in the cells of the lesions.** A few bronchial epithelial cells were pSMAD2 positive in WT mice (**A**). In the lesion of LXRαβ^-/-^ mouse lung, most of the cells were pSMAD2 nuclear-positive (**A**). Very few bronchial epithelial cells were SMAD4 positive in WT mice (**B**). Most of the cells in the lesion of LXRαβ^-/-^ mouse were SMAD4 positive (**B**). The number of cells that expressing pSMAD2 and SMAD4 was significantly higher than that in WT mice (**C**). RNA-seq data showed that in LXRαβ^-/-^ mouse lung there was a significant upregulation of TGFβ1, TGFβR1, and TGFβR2 with a downregulation of TGFβR3 in mRNA level (**D**). (Scale bars in A, B, 50 μm. #, P<0.05)

In order to investigate whether activated macrophages promote the lesion through transforming growth factor β (TGFβ) signaling, sections were stained for pSMAD2 and SMAD4. We found that compared with bronchial epithelial cells in WT mice, the cells in the lesion of LXRαβ^-/-^ mice expressed significantly higher levels of pSMAD2 (Fig. 4 A, C) and the co-SMAD, SMAD4 (Fig. 4 B, C). In LXRαβ^-/-^ mouse lungs, RNA-Seq data further confirmed the enhanced TGFβ signaling as there was upregulation of TGFβ1, TGFβR1, TGFβR2 with a downregulation of TGFβR3 (Fig. 4 D). There was no loss of PTEN and no increased pAKT as evidenced by PTEN nuclear-positive staining and absence of pAKT staining (Fig. S4 Ai, Aii). There was no detectable Wnt3a or pEGFR and β-catenin weakly positive in the cell membrane (Fig. S4 B, C). E-cadherin-positive staining and absence of vimentin staining confirmed that, even though TGFβ signaling was enhanced, there was no epithelial-to-mesenchymal transition (EMT) in the lesions (Fig. S4 D).

### Upregulation of MMPs in macrophages

**Figure 5:**
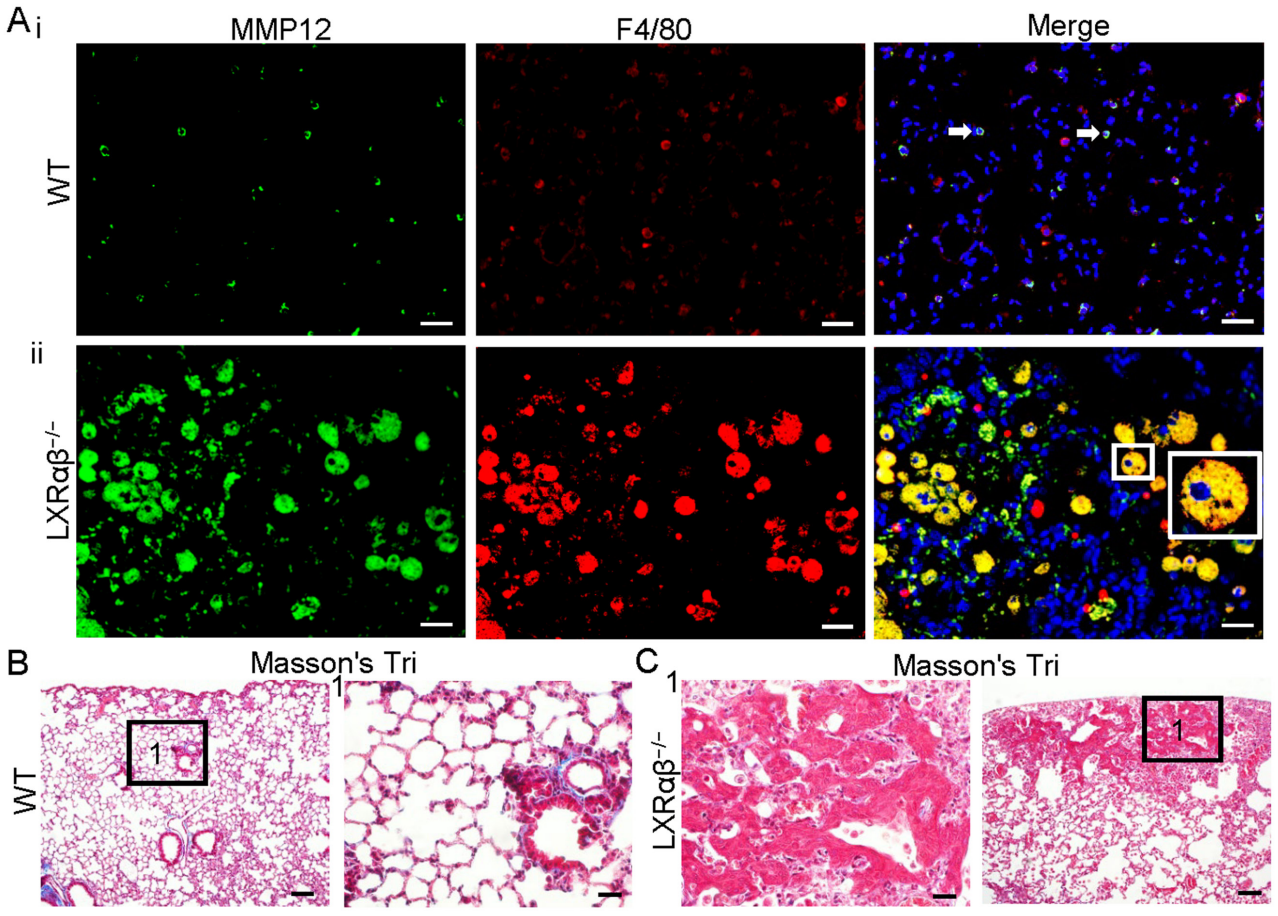
** Upregulation of MMP12 in macrophages with less collagen in LXRαβ^-/-^ mouse lung.** Double immunofluorescence staining for MMP12 (green) , F4/80 (red) and DAPI (blue) on WT mouse lung(**Ai**) in LXRαβ^-/-^ mouse lung (**Aii**). The MMP12 was expressed in a few macrophages in WT mouse lung (white arrows). Compared to WT mice there was a marked induction in the expression of MMP12 in macrophages of LXRαβ^-/-^ mouse lung (**Aii**). Inserted high magnification pictures in Aii show a macrophage co-expressed MMP12 and F4/80. Masson's Trichrome staining revealed a collagen layer surrounding the borehole of WT mice (**B**). No collagen in or around the lesion was found in the LXRαβ^-/-^ mouse lung (**C**). (Scale bars in A, 50 μm; Scale bars in B, C, 200 μm and 50 μm.)

Overproduction of several MMPs by macrophages are known to promote cancer progression 39. As assessed by RNA-seq, MMP12, MMP19, MMP8 and MMP14 were upregulated by 60-fold, 14-fold, 5-fold and 3-fold, respectively (Table S1). MMP12 and F4/80 double immunofluorescence staining confirmed the expression of MMP12 protein. Compared to WT mice (Fig. 5 Ai), there is a marked upregulation in the expression of MMP12 in macrophages of LXRαβ^-/-^ mouse lung (Fig. 5 Aii). In the lung of WT mice there was a collagen layer surrounding the borehole (Fig. 5 B). In LXRαβ^-/-^ mice there is no collagen in or around the lesion (Fig. 5 C). Compared to the WT mouse lung the expression of MMP8 and MMP14 was also upregulated in LXRαβ^-/-^ mouse lung (Fig. S5).

### Increased M1 and M2 polarization of macrophage and immune escape in LXRαβ^-/-^ mouse lung

**Figure 6:**
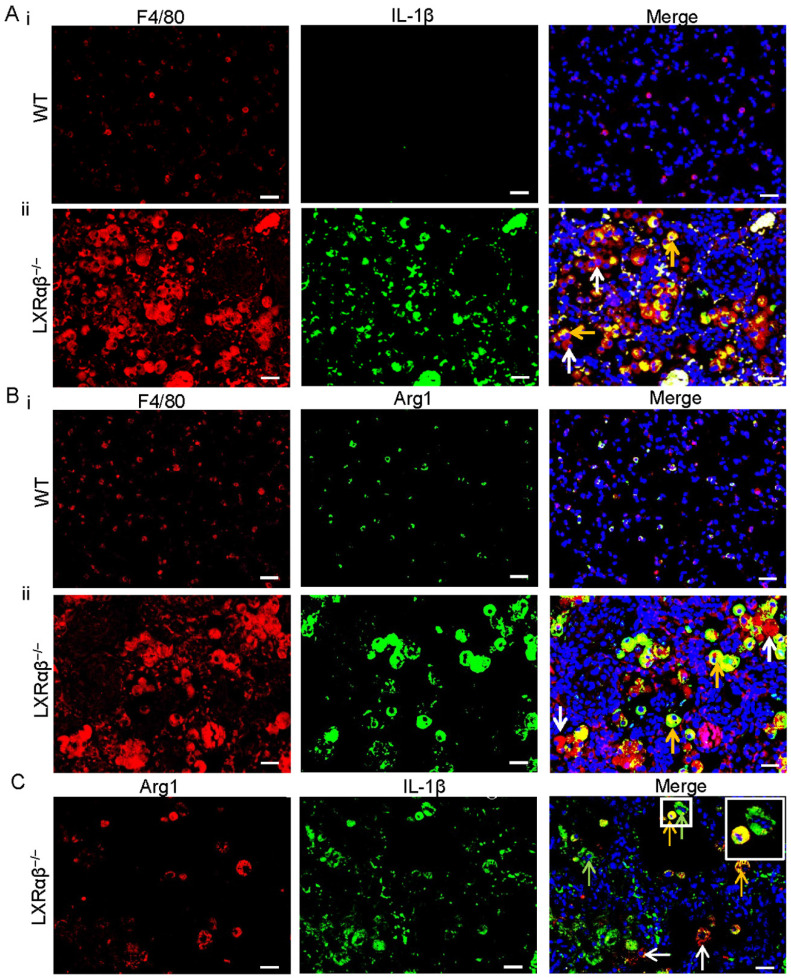
** Increased IL-1β (M1-macrophages) and arginase 1(M2-macrophages) expression in macrophages in LXRαβ^-/-^ mouse lung.** Double immunofluorescence staining for F4/80 (red), IL-1β (green) , and DAPI (blue) on WT mouse lung(**Ai**) and LXRαβ^-/-^ mouse lung (**Aii**). The expression of IL-1β was not detected by immunofluorescence in macrophages in WT mouse lung (**Ai**). The expression of IL-1β was a markedly induced in macrophages of LXRαβ^-/-^ mouse lung (**Aii**). Orange arrows showed that macrophages expressed IL-1β, white arrows showed that macrophages did not express IL-1β. Double immunofluorescence staining for F4/80 (red), Arg 1 (green) , and DAPI (blue) on WT mouse lung(**Bi**) and LXRαβ^-/-^ mouse lung (**Bii**). Arg 1 was expressed in some macrophages in WT mouse lung at a low level (**Bi**). The expression of Arg 1 was upregulated in macrophages of LXRαβ^-/-^ mouse lung (**Bii**). Orange arrows showed that macrophages expressed Arg 1, white arrow showed macrophages did not express Arg 1. Double immunofluorescence staining for Arg 1 (red), IL-1β (green) , and DAPI (blue) on LXRαβ^-/-^ mouse lung (**C**). Macrophage of LXRαβ^-/-^ mouse lung were both Arg 1 and IL-1β positive (orange arrows), Arg 1 positive (green arrows), or IL-1β positive (red arrows) (**C**). Inserted higher magnification picture in C indicated a macrophage expressed Arg 1 and IL-1β (orange) and a macrophage expressed only Arg 1(green). (Scale bars in A-C, 50 μm.)

RNA-Seq data revealed that genes for M1 macrophage (Nos2, CD86, IL-1β etc.) and genes for M2 macrophage (Arg1, Csf1r, pdcd1lg2 etc.) were both upregulated in the LXRαβ^-/-^ mouse lung (Table S2). To further investigate the pattern of macrophage polarization we used double immunofluorescence staining to localize IL-1β/F4/80, arginase 1(Arg1)/F4/80 and Arg1/IL-1β. IL-1β was not expressed in macrophages of WT mouse lungs (Fig. 6 Ai) but was strongly expressed in macrophages of LXRαβ^-/-^ mouse lung (Fig. 6 Aii). Macrophages in WT mouse lung expressed low level of Arg1 (Fig.6 Bi) but Arg1 was well expressed in macrophages in LXRαβ^-/-^ mouse lung (Fig. 6 Bii). Arg1/IL-1β double staining demonstrated that some macrophages in the lung of LXRαβ^-/-^ mouse can be both Arg1 and IL-1β positive (Fig. 6 C). To analyze T cell infiltration, we stained for FOXP3 (Treg marker), CD3 (total T cells) and CD8 (cytotoxic T cells). The number of Tregs (Fig. S6 A, D) and total T cells (Fig. S6 B, D) in the lesion of LXRαβ^-/-^ mouse lung was higher than that in WT mouse lung. However, there were very few cytotoxic T cells in the lesion (Fig. S6 C, D).

### Alteration in morphology with downregulated expression of estrogen receptor β (ERβ) of epithelial cells

**Figure 7:**
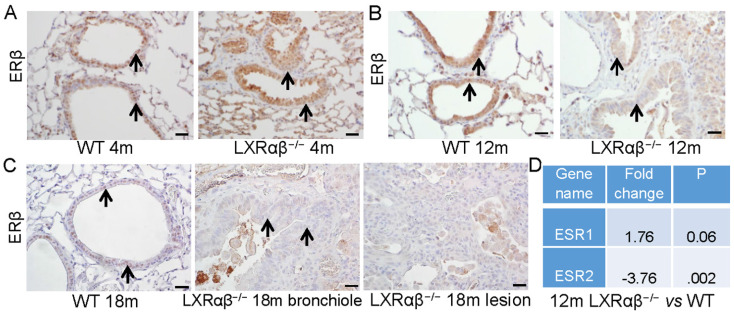
**Alteration in morphology with loss of the expression of ERβ in the epithelial cells of LXRαβ^-/-^ mice.** At the age of 4-mo there was a decrease in expression of ERβ in the epithelial cells on LXRαβ^-/-^ mouse lungs (**A**). At the age of 12-mo and 18-mo, expression of ERβ in the epithelial cells (**B, C**) and in cells in the lesion cells (**C**) of LXRαβ^-/-^ mice was deceased and there were morphological changes in the epithelial cells (longer cell bodies and piling up of cells in the epithelial layers) (**B, C**). RNA-Seq data showed that ERβ (ESR2) was significantly decreased in LXRαβ^-/-^ mouse lungs at the age of 12-mo (**D**). ERα (ESR1) was not changed (**D**). Black arrows indicated epithelial cells. (Scale bars in A-C, 50 μm.)

There were clear morphologic changes in the epithelial layer (longer cell bodies and piling up of epithelial cells) reminiscent of the LXRαβ^-/-^ mouse lungs (Fig. 7 A-C). ERβ has been previously shown to be essential for the maintenance of the extracellular matrix composition in the lung and loss of ERβ leads to abnormal lung structure 40. We found that in 4-month-old mice, there was a slight decrease of the expression of ERβ in the epithelial cells of LXRαβ^-/-^ mice (Fig. 7 A). However, at the age of 12-mo and 18-mo, expression of ERβ in the epithelial cells and in cells in the lesions of LXRαβ^-/-^ mice was markedly decreased (Fig. 7 B, C). RNA-Seq data confirmed that ERβ (ESR2), not ERα (ESR1) was significantly decreased in LXRαβ^-/-^ mouse lungs at the age of 12-mo (Fig. 7 D). Indicating that loss of ERβ in the lungs of older mice could have contributed to the morphological changes observed.

### Lower expression of LXRαβ or ERβ was correlated with shorter OS in LUSC patients with smoking history

Kaplan-Meier survival analysis was used to investigate whether the expression of LXRαβ correlated to OS in patients of lung squamous cell carcinoma (LUSC) related to smoking. The analysis showed that lower expression of either LXRα or LXRβ trends to a shorter OS in LUSC patients associated with smoking (Fig. S7 A, B) and lower expression of ERβ is also correlated with a shorter OS in these patients (Fig. S7 C).

## Discussion

Multiple phenotypes of LXRαβ^-/-^ mouse have been reported from 2002 (Table S3). The present study has uncovered a key role for LXRαβ in maintaining physiologic functions in the lung. The loss of LXRαβ led to the development of TTF-1/p63-positive NSCLC-like lesions and LUSC-like lesions. Traditionally lung cancers are categorized into two main histological groups, small cell lung carcinoma (SCLC) and non-small cell lung carcinoma (NSCLC), with the later accounting for 85% of the cases. Based mainly on the morphology of the transformed cells, NSCLC is further subcategorized into three categories: lung adenocarcinoma (LUAD), LUSC, and large-cell carcinoma (LCC) 41. LUAD originates from alveolar type II (AT2) epithelial cells or cells within bronchioalveolar duct junctions. They are TTF-1 positive and napsin A positive. LUSC is P40 positive, CK5/6 and P63 positive and is thought to develop from basal epithelial cells in airways 42. It is very uncommon that TTF-1 and P40 are expressed in the same cancer cells but in recent years some cases with TTF-1 and P40-positive NSCLC have been reported 43-46. TTF-1 is expressed in alveolar type II cells and in basal cells of the terminal respiratory unit and P40 is expressed in basal cells. The co-expression of TTF-1 and P40 suggests this rare type of NSCLC may origin from the basal cells of terminal respiratory unit. This "basal-type lung cancer" may be similar as the "basal-like" cancer of the breast i.e., triple-negative breast cancer (TNBC) 47. We have previous reported LUSC with adenomatous hyperplasia and squamous metaplasia in the lungs of 14-mo-old LXRαβ^-/-^ mice 34. In the present study we examined five 18-mo-old LXRαβ^-/-^ mice with their WT littermates. TTF-1/P40-positive hyperplasia and dysplasia (pre-cancer lesion) were found in all mouse lungs. TTF-1/P40-positive cancer-like lesion was identified in 3 mice. TTF-1/P40-positive lesions and the LUSC-like lesion coexisted in the lung of one mouse. To confirm the co-expression of TTF-1 and P40, two different TTF-1 antibodies, one P40 antibody, and one P63 antibody were used. As expected, the cells in basal layers of the terminal bronchial are TTF-1-positive and P40-positive. The cells in luminal layers are TTF-1 positive and P40- or P63-negative. Thus the antibodies we are using are specific for the intended targets. In the lesions of LXRαβ^-/-^ mice, the predominant staining pattern was that of LUSC with mild keratinization. Staining of serial slides in the lesion revealed both TTF-1-positive and P40- or P63-positive cells. TTF-1 and P40 double immunofluorescence staining further confirmed the co-expression of these two proteins.

To investigate the mechanisms contributing to development of the lesion, we analyzed gene expression in WT and LXRαβ^-/-^ mouse lungs. RNA-seq revealed that the most changed genes were related to macrophages. HE staining confirmed a substantial accumulation of macrophages in LXRαβ^-/-^ lung. CD68 staining showed in addition to an increase in number, there was a marked change in morphology of macrophages with cholesterol clefts and strong positive-staining by Oil-red-O indicating accumulation of cholesterol in macrophages in LXRαβ^-/-^ lung.

Lung tumor growth and metastasis is associated with enhanced TGFβ signaling, secretion of extracellular matrix degrading MMPs, blunting of T cell recruitment and promoting angiogenesis 48. TGFβ receptor-mediated signaling mainly depends on the phosphorylation and the nuclear translocation of SMAD proteins 49. In the LXRαβ lung lesions, cells expressed higher levels of pSMAD2 and SMAD4 bronchial epithelial cells in WT mice. RNA-seq confirmed an upregulation of TGFβ1, TGFβR1, TGFβR2 and a downregulation of TGFβR3. There was no immunohistochemical evidence for an increase of PTEN/pAKT, Wnt/β-catenin pathway. EGFR, a receptor tyrosine kinase, which is commonly upregulated in NSCLC was not present in the lesions.

Macrophages promote cancer invasion by secreting MMPs (MMP12, MMP8 and MMP14). Of these, MMP12 is the most abundant MMP. High MMP12 mRNA levels correlate with reduced survival in NSCLC and MMP12 promotes tumor growth in an orthotopic mouse model 50. MMP12 mRNA was upregulated by 60-fold in LXRαβ^-/-^ mice. The expression of MMP12 was markedly increased in almost all macrophages in the lungs of LXRαβ^-/-^ mice. Upregulation of MMP8 and MMP14 were also confirmed by IHC staining. These results indicate that in the absence of LXRαβ macrophages contribute development of the lesion by secreting MMPs.

Macrophage shape and functions are influenced by a variety of factors. Activated macrophages are classified as M1-like macrophages (pro-inflammatory macrophages) and M2-like macrophages (anti-inflammatory macrophages) 51. In general, activating LXRαβ by ligand promotes macrophage M2 polarization in inflammatory disease mouse models 52, 53. However, in the lung of LXRαβ^-/-^ mice macrophages exhibited characteristics of both M1 and M2 polarization (increase in both IL-1β and Arg1). Along with macrophage over-activation, we found in the lesion there was an increase in the number of regulatory T cells (Tregs). This increase may be caused by loss of LXRαβ in the Tregs directly or indirectly through the action of activated macrophages. Mice with conditional knockout LXRαβ in macrophages and Tregs are needed to further address this issue. With the increased Tregs and activate macrophages in the lesion, the cytotoxic T cells were prevented from infiltrating the lesion and this may explain how these abnormal cells escape immune surveillance.

In addition to alteration of macrophages there were some changes in epithelial cells. Disrupted cholesterol metabolism in the bronchial epithelial cells of LXRαβ^-/-^ mice was evident from the increase in Oil-red-O staining 34. In addition, there was a marked reduction in expression of expression of estrogen receptor β (ERβ), a nuclear receptor with tumor suppressive activities in epithelial cells 38, 40. Since ERβ also regulates the number of P63-positive basal cells in mammary gland and prostate 38, 54, its loss may be contribution to the increase in basal cells in the lung of LXRαβ^-/-^ mice.

CS is a leading cause of lung cancer; even secondhand smoke is associated with an increased risk of lung cancer 5. Many components of CS have been identified as carcinogens 55. CS was shown to induce the activation of lung macrophages with overexpression of MMP12 56, 57, which caused dysregulated homeostasis of the extracellular matrix and promoted the destruction of alveolar walls in the lungs. In patients of LUSC related to CS lower expression of either LXRα or LXRβ was associated with a shorter OS. Lower expression of ERβ in patients had a shorter OS too. Although mice were not exposed to CS in the present studies, our data suggest that there is an association between CS and repression of LXRαβ signaling.

In conclusion, our data suggest a key role of LXRαβ in maintaining the physiologic functions of the lung. Loss of LXRαβ over the long term caused the development of TTF-1/P63-positive NSCLC-like lesions and LUSC-like lesions. Since CS reduces expression of LXRαβ in the lung, we have introduced one additional mechanism through which CS leads to development of lung cancer. The study adds LXR agonists to the list of agents that may have a role in treatment of lung cancer.

## Supplementary Material

Supplementary figures and tables.

## Abbreviations

LXRαβ: Liver X receptors
ERβ: estrogen receptor β
CS: Cigarette smoke
NSCLC: non-small-cell lung cancer
LUSC: Lung squamous cell carcinoma
LUAD: Lung adenocarcinoma
LCC: Large-cell carcinoma
TTF-1: Thyroid transcription factor -1
TNBC: Triple-negative breast cancer
TGFβ: Transforming growth factor β
EGFR: epidermal growth factor receptor
MMPs: Matrix metalloproteases
FOXP3: Forkhead box protein P3
Tregs: Regulatory T cells
