# Supplementary material for: Liver X receptors and estrogen receptor β, two players in a rare subtype of NSCLC
Source: Int J Biol Sci. 2023 May 29;19(9):2848–59. doi: 10.7150/ijbs.85164 (PMC10266082; doi:10.7150/ijbs.85164)
Supplement: Supplementary file 1 — Supplementary figures and tables. [file ijbsv19p2848s1.pdf]

Supplementary Materials

Liver X receptors and estrogen receptor β, two players in a rare subtype of NSCLC

Wanfu Wu[1], Mozhgan Sarhadi[1], Xiaoyu Song[1], Jingling Xue[2], Yubing Dai[1], Jan-Ake Gustafsson[1]

1, Center for Nuclear Receptors and Cell Signaling, Department of Biology and Biochemistry, University of Houston, Houston, TX 77204, USA.

2, Department of Medical Microbiology, School of Basic Wuhan University, Wuhan, Hubei 430071, China

Correspondence:

WF Wu: Center for Nuclear Receptors and Cell Signaling, Department of Biology and Biochemistry, University of Houston, Houston, TX 77204, USA. Tel: 001-832-842-8805; Email: wwu16@central.uh.edu

JA Gustafsson: Center for Nuclear Receptors and Cell Signaling, Department of Biology and Biochemistry, University of Houston, Houston, TX 77204, USA. Tel: 001-832-842-8803; Email: jgustafsson@uh.edu

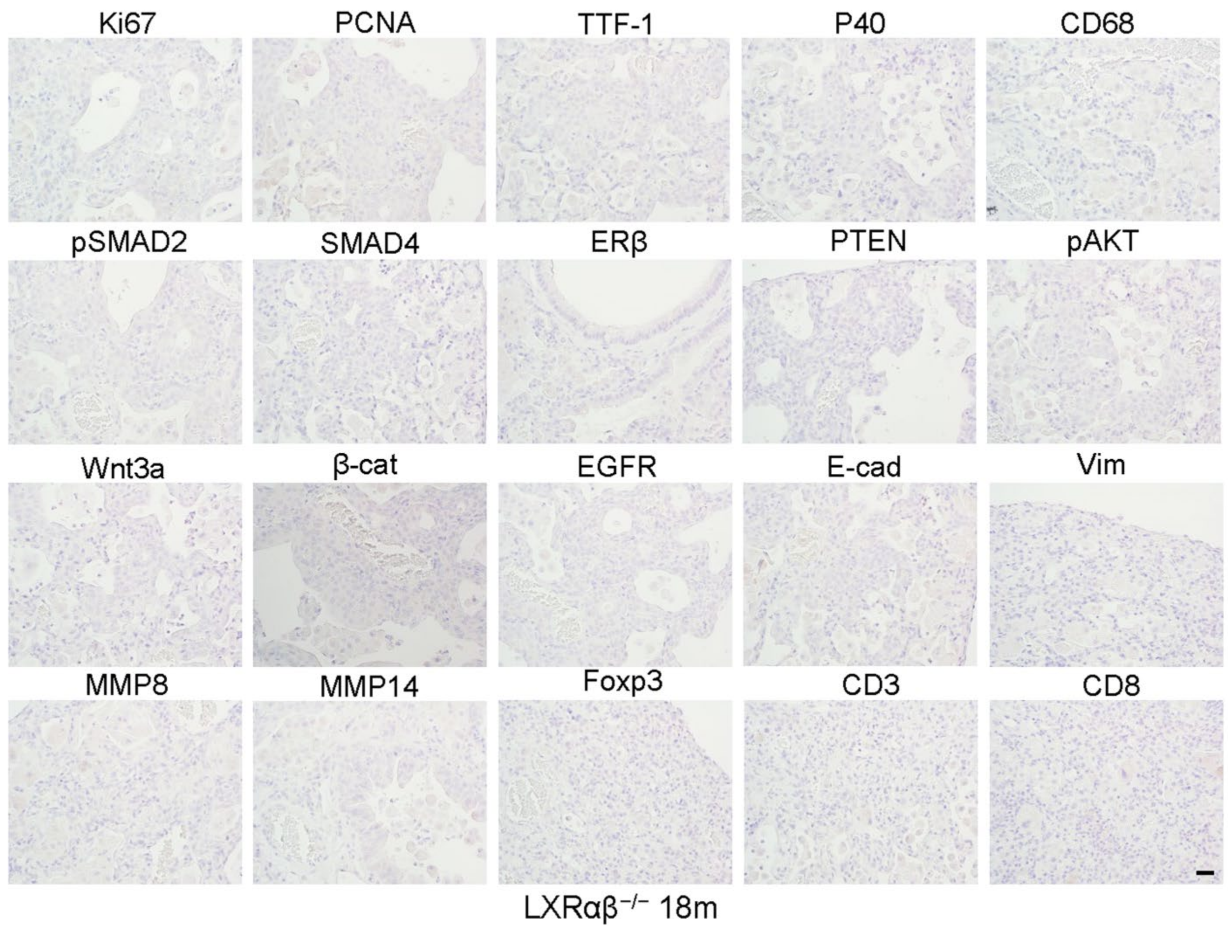

Fig. S1. Negative controls for the antibodies used in the study.

The omission of each primary antibody was used as negative control. (Scale bars, 50 µm)

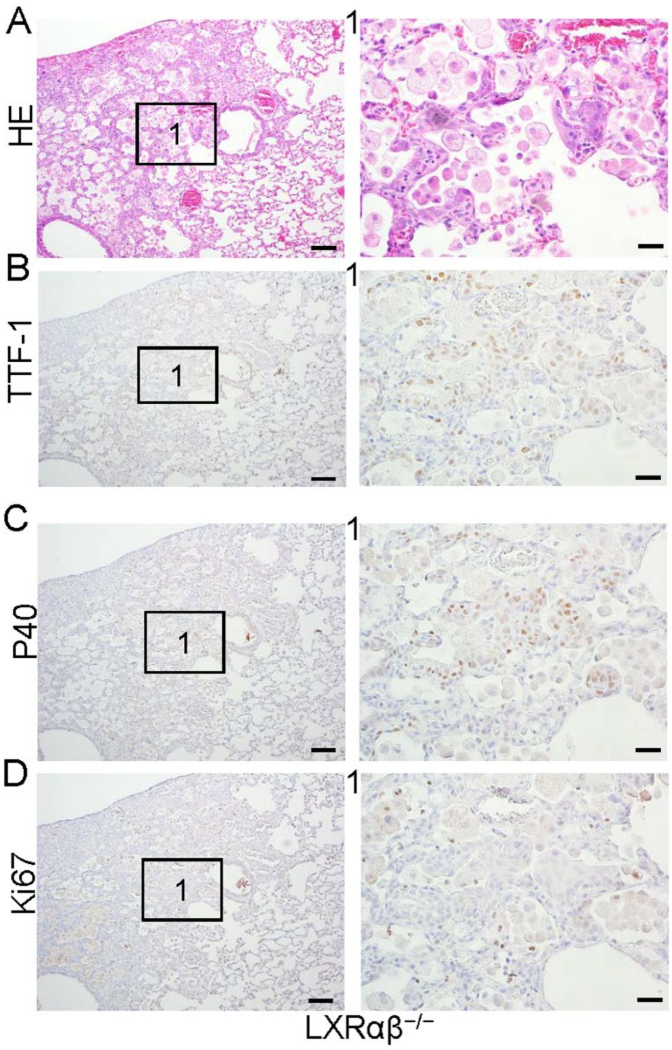

Fig. S2. Dysplasia of TTF-1/P40-positive cells in LXRαβ$^{-/-}$ mouse lung.

HE staining showed metaplasia in 18-month-old LXRαβ$^{-/-}$ mouse lung (**A**). TTF-1 and P40 staining on serial sections showed that TTF-1 and P40 were co-expressed in cells (**B, C**). Sparse Ki67 staining indicated these cells were not highly proliferating (**D**). (Scale bars in A-D, 200 μm and 50 μm)

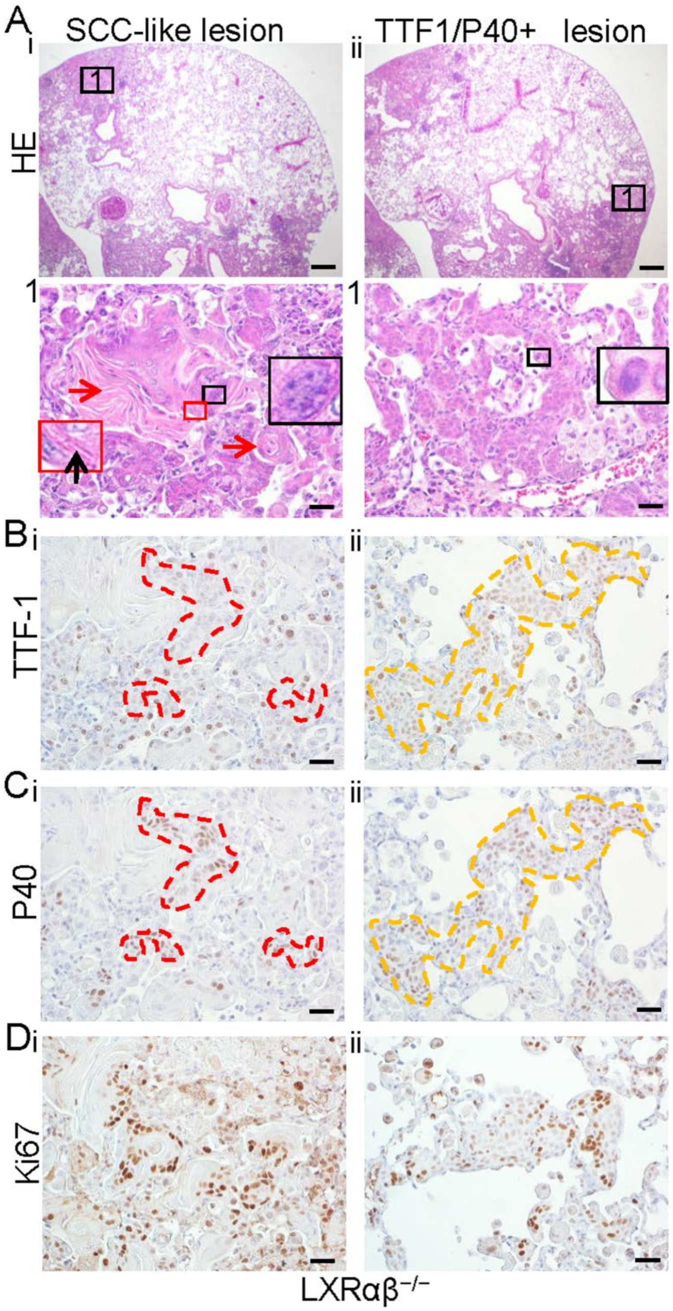

Fig. S3. The coexistence of typical SCC-like lesion and TTF-1/P40-positive lesions in LXRαβ$^{-/-}$ mouse lung.

HE staining demonstrated keratinization (red arrows), intercellular bridges (inserted picture with black arrow) and atypical mitosis (insert picture with black line) (**Ai**) accompanied with TTF-1 negative or very weakly-positive (**Bi**, irregular shapes with red dashed line), and P40-positve (**Ci**, irregular shapes with red dashed line) Ki67 positive (**Di**) for the typical SCC-like lesion. HE

staining showed atypical mitosis (insert picture in **Aii**1) without keratinization and intercellular bridges in TTF-1/p63-positive lesion (**Aii**). TTF-1 and P40 staining indicated these cells co-expressed TTF-1 and P40. (**Bii**, **Cii,** irregular shape with yellow dashed line) with positive staining for Ki67 (**Dii**). SCC: squamous cell carcinomas. (Scale bars in A, 500 μm and 50 μm; Scale bars in B-D, 50 μm)

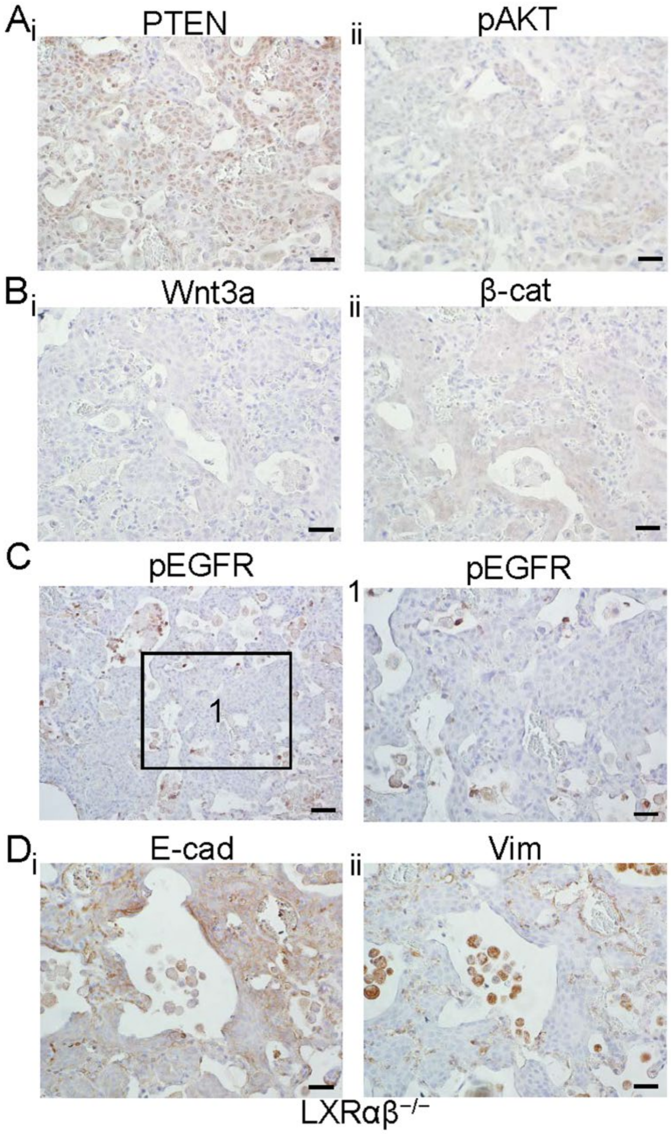

Fig. S4. Expression of PTEN/pAKT, Wnt3a/β Catenin, pEGFR, E-cadherin and vimentin in the lesion.

PTEN was well expressed in the abnormal cells (**Ai**) with very low expression of pAKT (**Aii**). There was very low expression of Wnt3a and β-Catenin (**Bi, Bii**). Expression of pEGFR was found on the cell membrane of macrophages not on the cancer-like cells (**C**). E-cadherin was well expressed on the cancer-like cells, but vimentin was not (**Di, Dii**). β-Cat: β-Catenin; E-cad: E-cadherin; Vim: vimentin. (Scale bars in A,B,D 50 µm; Scale bars in C, 100 µm and 50 µm)

Table S1: The comparison of genes for MMPs and TIMPs

Gene name	Log2 Fold change	P
Mmp12	60.34	0.003
Mmp19	14.17	0.0001
Mmp8	5.18	0.009
Mmp25	3.60	0.0999
Mmp14	3.39	0.0003
Mmp13	3.33	0.0001
Mmp9	-1.99	0.008
Timp1	8.12	0.0006
Timp2	1.16	0.585
Timp3	-1.02	0.902
Timp4	-1.72	0.143

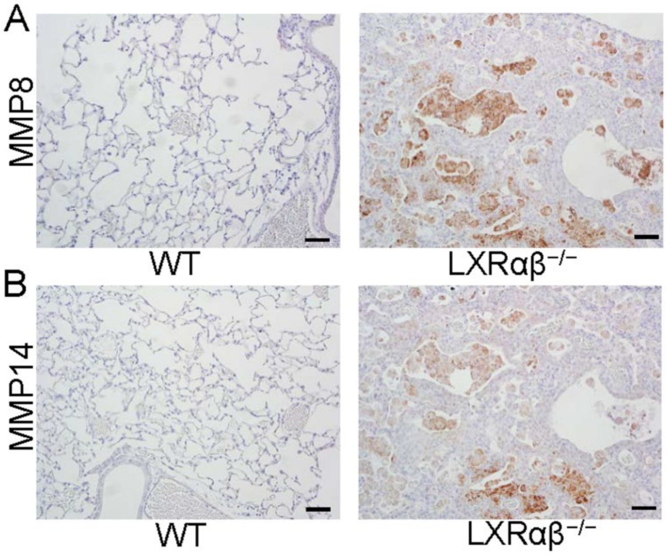

Fig. S5. Upregulation of MMP8 and MMP14 in macrophages in LXRαβ$^{-/-}$ mouse lung. Compared to WT mice there was a marked induction in the expression of MMP8 (**A**) and MMP14 (**B**). (Scale bars in A,B, 100 μm)

Table S2: The comparison of genes in Macrophage polarization

Gene name	Log2 Fold change	P
**M1**		
**Nos2**	2.60	0.002
**Cd86**	2.39	0.009
**Il1b**	3.62	0.028
**Il6**	5.77	0.056
**Tlr2**	6.10	0.002
**Ccl2**	8.30	0.003
**M2**		
**Arg1**	20.37	0.204
**Csf1r**	1.68	0.046
**Cd163**	-1.56	0.442
**Pdcd1lg2**	4.65	0.034
**Retnla**	17.82	0.162
**Chil3**	98.31	0.054

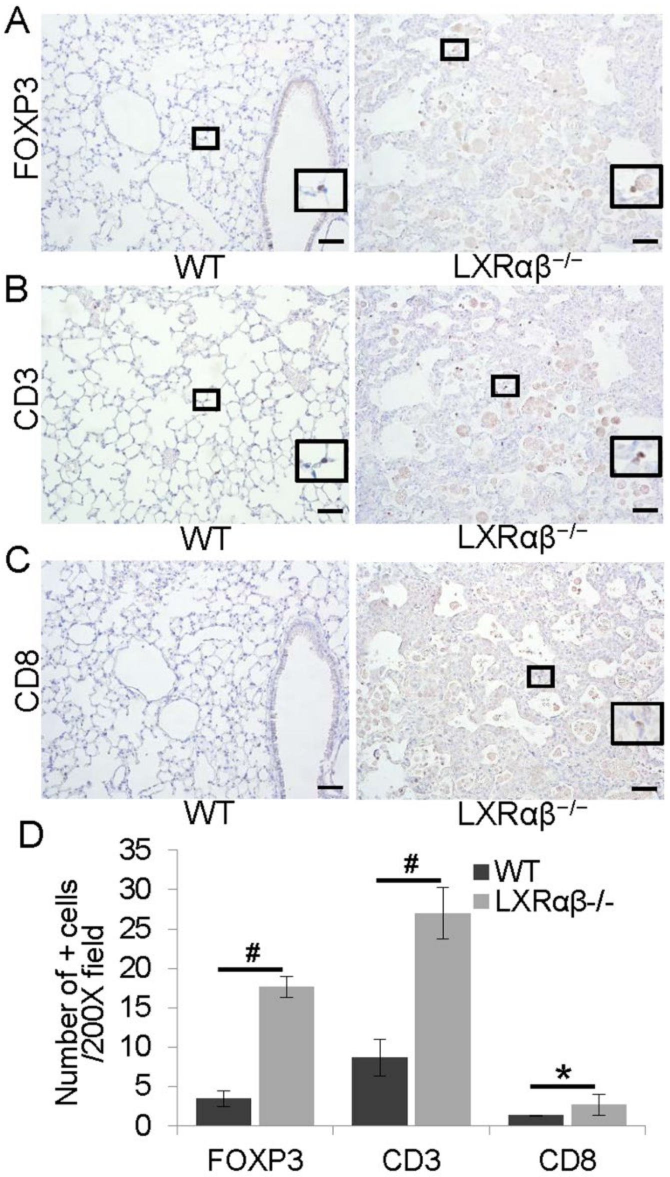

Fig. S6. **Increase in the number of FOXP3-positive and CD3-positve cells, but not CD8-positive cells in the lesion of LXRαβ$^{-/-}$ mouse lung.**

Compared to WT mice there was a significant increase in the number of FOXP3-positive Tregs (**A, D**) and CD3-positive T cells (**B, D**) in the lesion of LXRαβ$^{-/-}$ mouse lung. However, very few CD8-positive cytotoxic T cells were detectable in the lesion (**C, D**). Insert pictures were higher magnification. #: $p<0.05$; *: $p>0.05$. (Scale bars in -C, 100 μm)

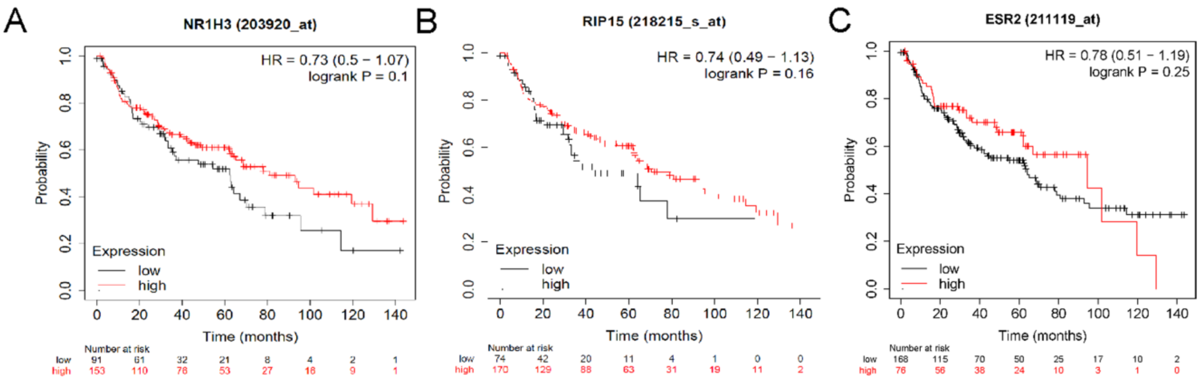

Median survival	
Low(mos)	High(mos)
62.3	80.9

Median survival	
Low(mos)	High(mos)
43.8	70.3

Median survival	
Low(mos)	High(mos)
64.1	94.5

Fig. S7. **The correlation between expression of LXRαβ or ERβ and OS in patients of LUSC related to smoking.**

The lower expression of either LXRα (A) or LXRβ (B) correlated to a shorter OS in LUSC patients and the lower expression of ERβ was also correlated with a shorter OS in these patients (C).

Table S3: The phenotypes of LXRαβ$^{-/-}$ mouse.

Organ/system	Phenotype	Reference
Spleen, lung, and artery	Foam cells in the spleen, lung, and arterial wall.	[1]
Liver	An increase in PB-mediated activation of Cyp2b10; Defection in hepatic lipid metabolism and resistance to obesity; Enhanced cholestatic sensitivity.	[2-4]
Kidney	Lower basal renin and blunted adrenergic response.	[5]
Testis	Infertile by alteration in testosterone synthesis, retinoid metabolism, and lipid metabolism.	[6]
Prostate	Stromal overgrowth and increased in epithelial cell proliferation.	[7]
Peripheral nervous system	Increased energy expenditure and weight loss.	[8]
Central nervous system	Defection in CSF production and structural integrity of choroid plexus.	[9]
Lung	Peripheral squamous cell lung cancer	[10]
Hematopoietic cells	Imbalance of hematopoietic populations with accelerated differentiation of the endothelial progenitor cells.	[11]
Thymus	Fatty, rapidly involuting thymus; Shrunken and prematurely immunoinhibitory peripheral T cell repertoire.	[12]

References

1. Schuster GU, Parini P, Wang L, Alberti S, Steffensen KR, Hansson GK, et al. Accumulation of foam cells in liver X receptor-deficient mice. Circulation. 2002; 106: 1147-53.
2. Gnerre C, Schuster GU, Roth A, Handschin C, Johansson L, Looser R, et al. LXR deficiency and cholesterol feeding affect the expression and phenobarbital-mediated induction of cytochromes P450 in mouse liver. J Lipid Res. 2005; 46: 1633-42.
3. Kalaany NY, Gauthier KC, Zavacki AM, Mammen PP, Kitazume T, Peterson JA, et al. LXRs regulate the balance between fat storage and oxidation. Cell Metab. 2005; 1: 231-44.
4. Uppal H, Saini SP, Moschetta A, Mu Y, Zhou J, Gong H, et al. Activation of LXRs prevents bile acid toxicity and cholestasis in female mice. Hepatology. 2007; 45: 422-32.
5. Morello F, de Boer RA, Steffensen KR, Gnecchi M, Chisholm JW, Boomsma F, et al. Liver X receptors alpha and beta regulate renin expression in vivo. J Clin Invest. 2005; 115: 1913-22.
6. Volle DH, Mouzat K, Duggavathi R, Siddeek B, Dechelotte P, Sion B, et al. Multiple roles of the nuclear receptors for oxysterols liver X receptor to maintain male fertility. Mol Endocrinol. 2007; 21: 1014-27.
7. Kim HJ, Andersson LC, Bouton D, Warner M, Gustafsson JA. Stromal growth and epithelial cell proliferation in ventral prostates of liver X receptor knockout mice. Proc Natl Acad Sci U S A. 2009; 106: 558-63.
8. Mansuy-Aubert V, Gautron L, Lee S, Bookout AL, Kusminski C, Sun K, et al. Loss of the liver X receptor LXRalpha/beta in peripheral sensory neurons modifies energy expenditure. Elife. 2015; 4.
9. Dai YB, Wu WF, Huang B, Miao YF, Nadarshina S, Warner M, et al. Liver X receptors regulate cerebrospinal fluid production. Mol Psychiatry. 2016; 21: 844-56.
10. Dai YB, Miao YF, Wu WF, Li Y, D'Errico F, Su W, et al. Ablation of Liver X receptors alpha and beta leads to spontaneous peripheral squamous cell lung cancer in mice. Proc Natl Acad Sci U S A. 2016; 113: 7614-9.
11. Rasheed A, Tsai R, Cummins CL. Loss of the Liver X Receptors Disrupts the Balance of Hematopoietic Populations, With Detrimental Effects on Endothelial Progenitor Cells. J Am Heart Assoc. 2018; 7.
12. Chan CT, Fenn AM, Harder NK, Mindur JE, McAlpine CS, Patel J, et al. Liver X receptors are required for thymic resilience and T cell output. J Exp Med. 2020; 217.
